# Microstructure and Mechanical Properties of Multilayered Ti-Based Bulk Metallic Glass Composites Containing Various Thicknesses of Ti-Rich Laminates

**DOI:** 10.3390/ma17133184

**Published:** 2024-06-28

**Authors:** Shifeng Lin, Lei Zhang, Rushan Lin, Zhengwang Zhu, Haifeng Zhang

**Affiliations:** 1 Department of Radiochemistry, China Institute of Atomic Energy, Beijing 102413, China; linshifeng06@163.com (S.L.); zhangll120619@163.com (L.Z.); 2 School of Metallurgy, Northeastern University, Shenyang 110819, China; hfzhang@imr.ac.cn

**Keywords:** multilayered composite, metallic glass composites, flexural mechanical properties, fracture toughness

## Abstract

In order to optimize the balance between strength and toughness, a series of multilayered Ti-based bulk metallic glass composites (BMGCs) with varying thicknesses of Ti-rich layers were successfully fabricated. The findings reveal that with an increase in the thickness of the Ti-rich layers, both the flexural yield strength and ultimate strength decreased from 2066 MPa and 2717 MPa to 668 MPa and 1163 MPa, respectively. Conversely, there was a noticeable increase in flexural strain. The fracture toughness of these multilayered Ti-based BMGCs decreased as the thickness of the Ti-rich layers increased; nevertheless, it stabilized at approximately 80 MPa·m^1/2^ when the thickness reached 100 μm. It was observed that a shift in the dominant deformation mode may be accountable for this phenomenon. These noteworthy characteristics suggest that adjusting the thickness of Ti-rich layers in multilayered BMGCs can effectively optimize mechanical performance, shedding light on the manufacturing of novel BMGCs with high performance.

## 1. Introduction

Bulk metallic glasses (BMGs) have emerged as a promising engineering material, owing to their exceptional properties such as high strength, high hardness, excellent wear resistance, *etc*. [1,2,3,4,5]. However, the inherent room-temperature brittleness of BMGs has hindered their widespread structural application. To overcome this challenge, a viable approach is the development of BMG composites (BMGCs) through the incorporation of crystalline phases [4,6,7,8]. These crystalline phases are anticipated to facilitate shear band nucleation and hinder shear band propagation, thereby enhancing the plasticity of the BMGCs.

Among the various techniques employed in the fabrication of composite materials, *ex-situ* and *in-situ* strategies are notably prevalent [9,10]. The *in-situ* approach entails the precipitation of secondary dendrites from the glassy matrix to form BMGCs, achieved through careful adjustments in the alloy composition or cooling rates [9,11,12,13]. This *in-situ* methodology yields BMGCs characterized by exceptional plasticity under tensile loading, owing to the perfect interface between dendrites and the glassy matrix. Conversely, *ex-situ* BMGCs are prepared by introducing foreign phases, such as fibers, wires, and particles [14,15,16,17,18], which effectively initiate and propagate shear bands, thereby facilitating plastic deformation and thus significantly enhancing compressive plasticity rather than tensile plasticity.

However, the trade-off between the mechanical properties such as strength, ductility, and fracture toughness remains a significant challenge for both *in-situ* and *ex-situ* BMGs or BMGCs, acting as the proverbial ‘Achilles heel’ and limiting their industrial application. To address this dilemma, we developed an *in-situ* and *ex-situ* hybrid strategy to design multilayered BMGCs with a multiscale hierarchical structure, as introduced in our previous works, which demonstrate highly desirable comprehensive mechanical properties [19,20,21,22]. The multilayered BMGCs were prepared by assembling alternating pure Ti layers and metallic glass ribbons of identical thickness, heating to the melting temperature of the amorphous alloy, and subsequently water quenching. Apart from the mechanical properties, the cost and density are also important factors to consider for the commercialization of the BMGs or BMGCs. In the Ti-based multilayered BMGCs, increasing the Ti content by increasing the thickness of Ti layers in the assembling process is beneficial to further reduce the density and cost of the resultant BMGCs. It is widely acknowledged that crystalline phases play a critical role in enhancing the mechanical properties of BMGCs [23,24,25]. Therefore, significant efforts have been directed towards optimizing BMGC mechanical properties by adjusting the sizes, spacing, shape, distribution, and volume fraction of these crystalline phases. Among these, modifying the volume fraction effectively tunes the mechanical properties of BMGCs. In our previous work [19], we observed that reducing the thickness of pure Ti layers in the multilayered BMGC resulted in the formation of an α-phase with the volume fraction of ~18%. However, the existence of the α-phase was shown to be crucial to the final mechanical properties. In this study, multilayered Ti-Based BMGCs containing various thicknesses of Ti-rich laminates were prepared using pure Ti layers with different thicknesses. The corresponding microstructural evolution and mechanical properties, including flexural properties and fracture toughness, were studied.

## 2. Materials and Methods

**Figure 1:**
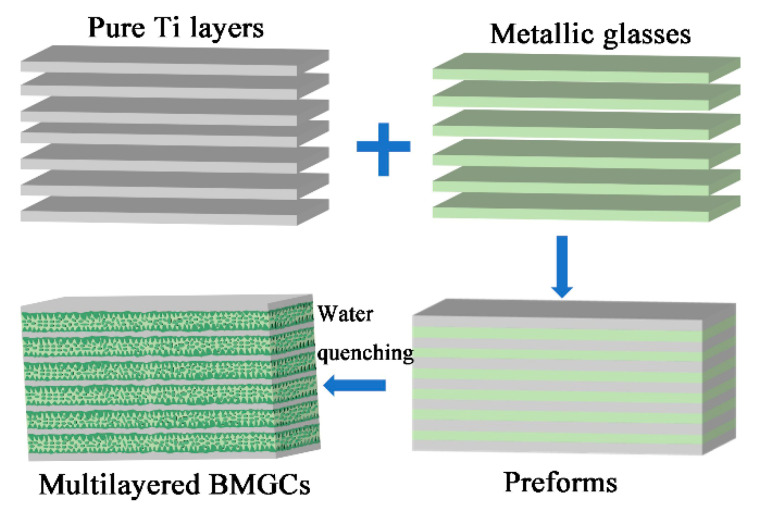
Process diagram of preparing bulk metallic glass composites (BMGCs) with layers of varying thickness.

The Ti_31.16_Zr_28.69_Ni_5.04_Cu_8.55_Be_21.56_Mo_5_ (at.%) BMG ingots were prepared by arc melting a mixture of pure elements (purity wt% ≥ 99.9%) under a Ti-gettered high-purity argon atmosphere. The process of preparing bulk metallic glass composites (BMGCs) with layers of varying thickness is shown in Figure 1. The BMG ingots were re-melted at least four times to ensure compositional homogeneity. The metallic glass (MG) ribbons of about 50 μm in thickness and 10 mm in width were produced by a melt spinning method. The commercially pure Ti layers of 50 μm, 80 μm, 100 μm, 150 μm, and 200 μm in thickness were cut into the same dimensions as the MG ribbons. These pure Ti layers and MG ribbons were ultrasonically cleaned in ethanol. The pure Ti layers and MG ribbons with different thicknesses were then alternately stacked to create preforms with a thickness of 10 mm and length of 80 mm. The preparation process of the BMGCs from our previous works was used as the reference [19,20,21,22]. The BMGCs prepared with Ti layers of 50 μm, 80 μm, 100 μm, 150 μm and 200 μm in thickness are denoted as Ti50, Ti80, Ti100, Ti150 and Ti200, respectively.

**Figure 2:**
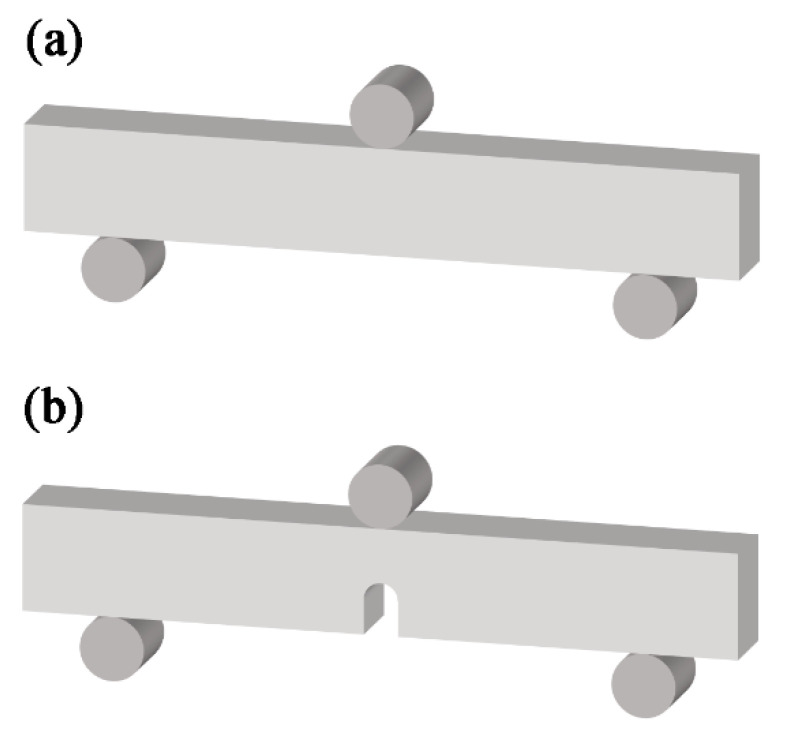
Schematic diagram of the test for three-point single-edge notched bend (**a**) and unnotched bend (**b**).

The microstructure of the as-prepared BMGCs were characterized by means of X-ray diffraction (XRD, Philips PW 1050, Cu-Kα; Philips, Eindhoven, The Netherlands) and scanning electron microscopy (SEM, Zeiss Supra 55; Zeiss, Jena, Germany). The three-point single-edge notched bend (SENB) and unnotched bend were measured using a universal testing machine (Instron 5582; Instron, Boston, MA, USA) with a constant displacement rate of 0.1 mm/min and the span of 20 mm, as seen in Figure 2. The sample dimensions of the three-point SENB and unnotched bend were as follows: *B* (thickness) = 2 mm, *W* (width) = 4 mm, and *L* (length) = 30 mm; and *B* = 2.5 mm, *W* = 5 mm, and *L* = 30 mm, respectively. The notches of the three-point SENB samples with a root radius of about 125 μm were made to a depth of 0.45 *W*–0.55 *W*. After the bending, the samples were observed by scanning electron microscopy (SEM).

## 3. Results and Discussion

**Figure 3:**
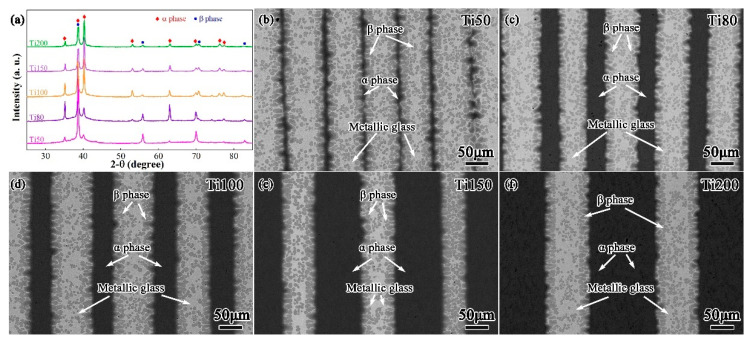
(**a**) XRD patterns of the as-cast multilayered BMGCs with different thicknesses of Ti layers; (**b**–**f**) SEM morphology of Ti50, Ti80, Ti100, Ti150 and Ti200 multilayered BMGCs, respectively.

Figure 3a shows the XRD patterns of the as-prepared multilayered BMGCs with various thicknesses of Ti-rich laminates. The XRD patterns demonstrate that these BMGCs are comprised of the α-phase, β-phase, and MG matrix. Figure 3b–f show the SEM images of these BMGCs. Apparently, an alternate distribution with the Ti and glassy layers is observed, conforming to the anticipated designed structure. It can be seen that the residual pure Ti (black) after the dissolution increases in thickness as the thickness of the Ti layer increases, which is highly expected. The α-phase (dark regions) and β-phase (gray regions) are dispersively distributed in the glassy matrix (the remaining bright regions). The interfacial reaction between the reinforcement phase and the glassy matrix has always been a fatal problem for ex situ BMGCs [14,26,27]. However, this defect does not appear in the present multilayered BMGCs. It is evident from Figure 3b–f that there are no porosities and microcracks in the overall cross-section of the multilayered BMGCs, which reveals the relatively well-bonded characteristic that the interfaces between the Ti layers and the glassy matrix obtained during the preparation process. The microstructures of these multilayered BMGCs are also consistent with those of the Ti-based multilayered BMGCs previously reported [20,21].

**Figure 4:**
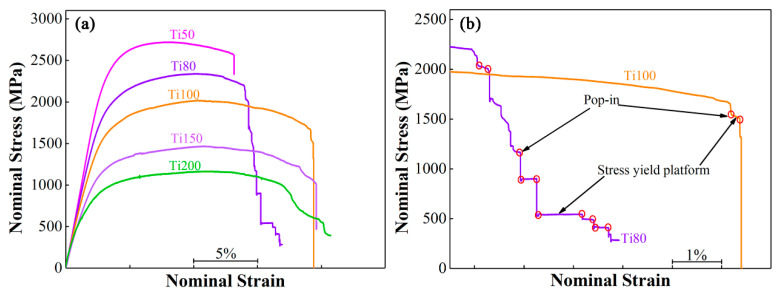
(**a**) The flexural nominal stress–strain curves of the multilayered BMGCs with different thickness of Ti layers under three-point single-edge unnotched bending conditions; (**b**) Local magnification flexural nominal stress–strain curves of (**a**).

The stress–strain curves under the three-point bending condition are shown in Figure 4a. It is apparent that all the BMGCs exhibit a work-hardening behavior after yielding, followed by a gradual decline in the stress. The yielding strength and ultimate strength are summarized in Table 1 for all the current BMGCs. It is clearly shown that the yielding strength and ultimate strength decrease considerably from 2066 MPa and 2717 MPa for Ti50 to 668 MPa and 1163 MPa for Ti200 as the thickness of the Ti-rich layers increases. Among them, the Ti150 multilayered BMGCs exhibit the most reduction in yield strength and ultimate strength, reaching ~312 MPa and ~372 MPa, respectively, compared with the Ti100 multilayered BMGCs.

In contrast, the bending strain considerably increases. This phenomenon is obviously attributed to the volume fraction effect where the fraction volume of the softer Ti-rich layers increases while the strong glassy matrix decreases, resulting in the trade-off between strength and plasticity.

**Figure 5:**
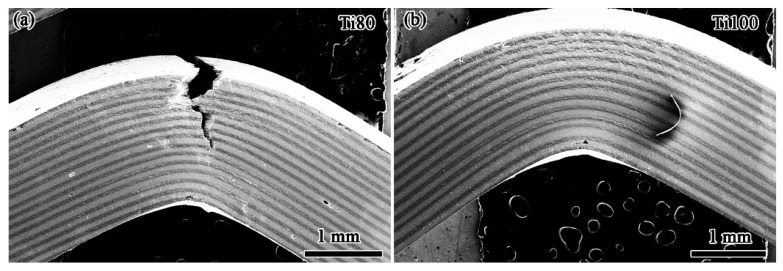
The morphologies of Ti80 (**a**) and Ti100 (**b**) multilayered BMGC after the flexuous failure under single-edge unnotched three-point bending conditions.

Interestingly, it is observed from Figure 4a,b that the multilayered BMGCs exhibit “Pop in” points and elongated stress yielding platforms as the thickness of the Ti layers increases to 80 μm, which will be beneficial to improving fracture toughness and fracture work as they can effectively change the propagation paths of the cracks [28]. In comparison, the Ti80 multilayered BMGC possesses more “Pop in” points and elongated stress yielding platforms than those of the other multilayered BMGCs. The multilayered BMGCs with the Ti layers’ thickness not exceeding 80 μm fails by way of crack propagation, as seen in Figure 3a, which reveals that the deformation of the multilayered BMGCs is crucially dominated by the strong glassy matrix. The Ti80 multilayered BMGC exhibits a zigzag and tortuous main crack propagation path unlike in the representative BMGs, where the crack propagates in a straightforward manner, in a direction that is nearly perpendicular to the loading stress, and without any plastic deformation [29,30,31,32]. The moderate strength and plasticity of the Ti80 multilayered BMGC benefits from the interaction between the glassy matrix and the crystalline phases. A large number of apparent plastic deformation features are detected around the main crack (Figure 5a), indicating that the ductile β-phase embedded in the glassy matrix and the continuous Ti layers collectively delay the shear bands during the process of loading from evolving into further cracking such that the Ti80 multilayered BMGC becomes endowed with a moderate flexuous mechanical property. In contrast, the multilayered BMGCs with Ti layers reaching 100 μm in thickness did not crack open or fracture after failure, as seen in the Ti100 multilayered BMGC in Figure 5b, which suggests that the deformation of the multilayered BMGCs is dominated by the ductile Ti layer. This matches with the phenomenon that the stress decreases while the strain increases in Figure 4b. It is observed that multiple shear bands appear on the glassy matrix, originating from the propagation of the primary shear band which is hindered by the Ti layers. Evidently, the plastic deformation of the Ti layers in the Ti100 multilayered BMGC is more severe than that of the Ti80 multilayered BMGC. Therefore, the compromise between strength and plasticity for the Ti100 multilayered BMGC occurs due to the higher volume fraction of crystal Ti layers.

**Figure 6:**
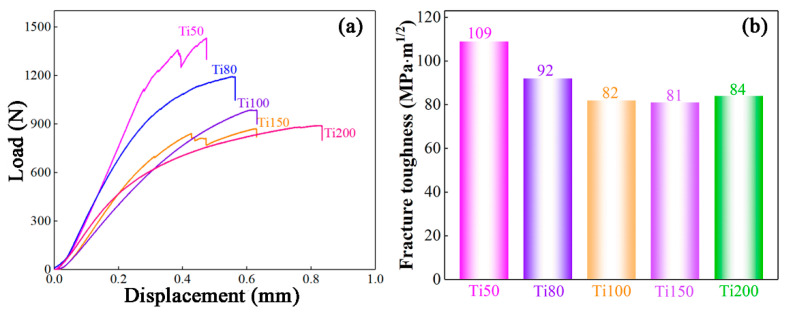
The load–displacement curves (**a**) and the fracture toughness (**b**) of the multilayered BMGCs with different thickness of Ti layers under three-point single-edge notched bending conditions.

The present multilayered BMGCs are fractured in a nonlinear-elastic mode under SENB testing, as seen in Figure 6a. Accordingly, the fracture toughness is calculated using the standard mode I J–K equivalence relationship [33,34] as follows:

(1) $$K_{J}=\sqrt{JE^{\prime }}=\sqrt{1.9A_{tot}E/(1-v^{2})Bb}$$

where $E^{\prime }=E/(1-v^{2})$, E=88 GB and v=0.37 are Young’ modulus and the Poisson’s ratio, respectively. *J* is the *J*-integral and a function of the crack extension, ∆*a*. *A_tot_* is the total area under the standard force–displacement curve, including the elastic and plastic contributions to the fracture energy. *B* is the thickness of the specimen, and *b* is the uncracked ligament width (i.e., *b* = *W* − ∆*a*).

**Figure 7:**
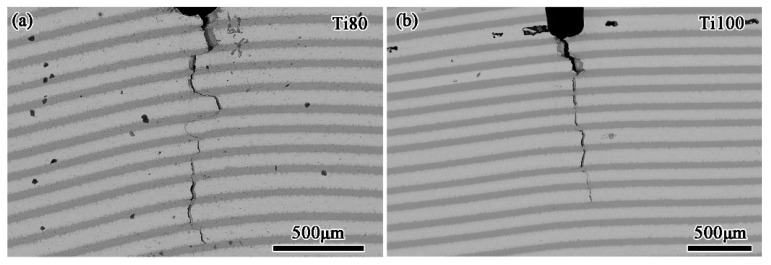
SEM micrographs of the fracture surfaces of the Ti80 (**a**) and Ti100 (**b**) multilayered BMGCs after failure under three-point single-edge notched bending conditions.

The calculated fracture toughness for the present multilayered BMGCs, according to Equation (1), is shown in Figure 6b. The fracture toughness decreases gradually with the thickness of the Ti-rich layer, which results from the softer Ti layers increasing in volume fraction. It is worth noting that the fracture toughness tends to be stable at ~80 MPa·m^1/2^ as the Ti layers decrease to 100 μm in thickness. This result further explains that the deformation of the multilayered BMGCs is decisively controlled by the plastic Ti layers with a thickness of greater than 100 μm. In order to visually illustrate the fracture characteristics, the Ti80 and Ti100 multilayered BMGCs are taken as examples. The morphology of the fracture surfaces of the Ti80 and Ti100 multilayered BMGCs after the notches of the three-point SENBs are examined by SEM is described in Figure 7. The unstable crack growth causes a catastrophically brittle fracture in the BMGC, leading to low fracture toughness. However, this predicament can be averted by introducing ductile phases, as they can reduce the driving force of the crack [34,35,36,37]. Consequently, the present multilayered BMGCs possesses high fracture toughness. From Figure 7a, the crack in the Ti80 multilayered BMGC propagates in parallel to the notch depth, but the path is significantly deflected. Compared to the Ti80 multilayered BMGC, the crack in the Ti100 multilayered BMGC is relatively straight, as seen in Figure 7b. This difference originates from the difference in the volume fraction of the crystalline phases [12,35,38,39]. The fracture toughness of the multilayered BMGCs is no longer significantly reduced as the thickness of the Ti layers increases to 100 μm, which may be responsible for the dominant deformation mode transformation.

## 4. Conclusions

The multilayered Ti-based bulk metallic glass composites (BMGCs) with different thicknesses of Ti-rich layers were successfully prepared by melting a preform of an alternating stack-up of Ti foils in a high-vacuum atmosphere. The multilayered Ti-based BMGCs exhibited a structure of Ti layers alternating with a glassy matrix. The Ti layers in the multilayered Ti-based BMGCs became thicker with the increase in each Ti layer’s thickness. The flexural strength decreased while the flexural strain increased with the Ti layers increasing. The yielding strength and ultimate strength decreased considerably from 2066 MPa and 2717 MPa for Ti50 to 668 MPa and 1163 MPa for Ti200 as the thickness of the Ti layers increased. However, the bending strain of the multilayered BMGCs considerably increased with the Ti layers increasing in thickness. The variation in the bending mechanical properties can be attributed to the volume fraction. Interestingly, the fracture toughness of the multilayered BMGCs decreased as the Ti layers became thicker. Nevertheless, the fracture toughness stabilized at ~80 MPa·m^1/2^, which may be responsible for the transformation of the dominant mode of deformation. For applications, the Ti50 multilayered BMGC would be optimal for achieving maximum strength, while the Ti200 multilayered BMGC would be preferable for obtaining the best ductile deformations. For a comprehensive balance of strength and ductility, the Ti100 multilayered BMGC appears to be the optimal choice as it combines high strength with significant ductile deformation.

## Figures and Tables

**Table 1:** The flexural mechanical properties of the multilayered BMGCs.

BMGCs	Ti50	Ti80	Ti100	Ti150	Ti200
Yield strength, σ_y_ (MPa)	2066	1754	1482	964	668
Ultimate strength, σ_u_ (MPa)	2717	2345	2025	1468	1163

## Data Availability

Data are contained within the article.
